# Comparative chloroplast genome and transcriptome analysis on the ancient genus *Isoetes* from China

**DOI:** 10.3389/fpls.2022.924559

**Published:** 2022-07-29

**Authors:** Yujiao Yang, Xiaolei Yu, Pei Wei, Chenlai Liu, Zhuyifu Chen, Xiaoyan Li, Xing Liu

**Affiliations:** ^1^State Key Laboratory of Hybrid Rice, Laboratory of Plant Systematics and Evolutionary Biology, College of Life Sciences, Wuhan University, Wuhan, China; ^2^Biology Experimental Teaching Center, School of Life Science, Wuhan University, Wuhan, China

**Keywords:** *Isoetes*, chloroplast genome, transcriptome, adaptive evolution, RNA editing

## Abstract

*Isoetes* is a famous living fossil that plays a significant role in the evolutionary studies of the plant kingdom. To explore the adaptive evolution of the ancient genus Isoetes from China, we focused on *Isoetes yunguiensis* (Q.F. Wang and W.C. Taylor), *I. shangrilaensis* (X. Li, Y.Q. Huang, X.K. Dai & X. Liu), *I. taiwanensis* (DeVol), *I. sinensis* (T.C. Palmer), *I. hypsophila_*GHC (Handel-Mazzetti), and *I. hypsophila_*HZS in this study. We sequenced, assembled, and annotated six individuals’ chloroplast genomes and transcriptomes, and performed a series of analyses to investigate their chloroplast genome structures, RNA editing events, and adaptive evolution. The six chloroplast genomes of *Isoetes* exhibited a typical quadripartite structure with conserved genome sequence and structure. Comparative analyses of *Isoetes* species demonstrated that the gene organization, genome size, and GC contents of the chloroplast genome are highly conserved across the genus. Besides, our positive selection analyses suggested that one positively selected gene was statistically supported in *Isoetes* chloroplast genomes using the likelihood ratio test (LRT) based on branch-site models. Moreover, we detected positive selection signals using transcriptome data, suggesting that nuclear-encoded genes involved in the adaption of *Isoetes* species to the extreme environment of the Qinghai-Tibetan Plateau (QTP). In addition, we identified 291–579 RNA editing sites in the chloroplast genomes of six *Isoetes* based on transcriptome data, well above the average of angiosperms. RNA editing in protein-coding transcripts results from amino acid changes to increase their hydrophobicity and conservation in *Isoetes*, which may help proteins form functional three-dimensional structure. Overall, the results of this study provide comprehensive transcriptome and chloroplast genome resources and contribute to a better understanding of adaptive evolutionary and molecular biology in *Isoetes*.

## Introduction

Lycopsids, the sister of the remaining vascular plants and an important bridge between non-vascular bryophytes and vascular plants, is a key group in evolution (Pryer et al., 2001), but it currently includes only three major lineages (Lycopodiaceae, Isoetaceae, and Selaginellaceae). These groups were widely distributed over the Carboniferous, being the dominant plants on earth (DiMichele et al., 2001). It was not until the end of the Carboniferous that these groups began to diminish due to dramatic changes in climate and environment (Liu et al., 2005). *Isoetes* is an ancient heterosporous lycopsids that occupies a unique position in plant evolution and there are ~200 extant species (Pigg, 1992). Phylogenetic analyses show that this genus is one of the earliest basal vascular plants, which can date back to the Devonian (Pigg, 2001; Pryer et al., 2001). The modern distribution of *Isoetes* is influenced by geographic variation (Liu et al., 2004), and they grow in a variety of habitats, including seasonal pools, intermittent streams, and high-altitude wetlands (Pfeiffer, 1922; Taylor and Hickey, 1992). To date, six *Isoetes* species have been reported in China: *I. yunguiensis* (Qing-Feng et al., 2002), *I. hypsophila*, *I. shangrilaensis* (Li et al., 2019), *I. taiwanensis* (DeVol, 1972), *I. sinensis* (Palmer, 1927), and *I. orientalis* (Hong et al., 2005). Species of the genus *Isoetes* are widely distributed in China, about 100–4,300 m above the sea level. However, it does not fit the hypothesis that the distribution of polyploids is more likely at high altitudes (Liu et al., 2004). *I. hypsophila* (2*n* = 22) inhabits altitudes above 4,000 m on the QTP, which is the youngest, largest, and highest plateau in the world, while *I. sinensis* (4*n* = 44) inhabits low-altitude environments in the Middle and Lower Yangtze Plain (MYP; Xing et al., 2002; Liu et al., 2004).

The QTP is characterized by low temperature, low oxygen, and strong radiation, which offers a unique extreme environment for studying adaptive evolution (Qiao et al., 2016a,b). Although the adaptive evolution of QTP has been studied previously in animals (Hao et al., 2019) and plants (Zhang et al., 2019; Guo et al., 2020), these studies are far from sufficient because different organisms adapt to high altitudes through multiple genetic routes (Hao et al., 2019). Notably, hitherto, no study of adaptive evolution has been conducted on *Isoetes* based on combined transcriptome and chloroplast genome analysis.

Chloroplasts are photosynthetic organelles that play an irreplaceable role in plant growth and development (Liu et al., 2012). Most chloroplast genomes have a circular structure ranging from 110 to 190 kb in size and consist of a relatively conserved quadripartite structure, including two Inverted Repeat (IR) regions, a Small Single Copy (SSC), and a Large Single Copy (LSC) region (Palmer, 1985; Green, 2011; Yu et al., 2019). Despite the chloroplast genome being relatively conservative in gene content, structure, and gene order (Shahzadi et al., 2020; Yu et al., 2021), numerous early evolutionary modifying mutational events frequently occur in the chloroplast genome, including inversions, contractions, substitutions, gene loss, duplications, and pseudogenes (Raubeson and Jansen, 1992a,b; Henriquez et al., 2020; Shahzadi et al., 2020). As the chloroplast is the center of photosynthesis, the study of the chloroplast genome is important for discovering the mechanisms of plant photosynthesis.

RNA editing is a post-transcriptional modification that changes nucleotide sequences of RNA by nucleotide insertions/deletions or transitions (Takenaka et al., 2013), this phenomenon occurs in different regions of the chloroplast genome such as protein-coding regions, introns, and tRNAs (Schallenberg-Rüdinger and Knoop, 2016; Stefan, 2016). Although RNA editing is not limited to protein-coding regions, it may play a fundamental role in these regions, potentially affecting species phenotype and evolution by maintaining the basic functions of genes. In protein-coding genes, it generally implies generating start/stop codons, restoring codons for amino acids, or removing internal stop codons (Schallenberg-Rüdinger and Knoop, 2016). In other regions, RNA editing performs different functions; for example, in tRNA, the processing of precursor RNA molecules may require editing, while in introns, editing events appear to be required for efficient splicing in some cases (Binder et al., 1994). RNA editing is considered to be an indirect repair mechanism for the correction of DNA mutations on the RNA level by converting specific cytidine to uridine (C-to-U) or uridine to cytidine (U-to-C; Chateigner-Boutin and Small, 2011; Ichinose and Sugita, 2016). In plants, the most common type of RNA editing in the chloroplast genome is C-to-U editing. In contrast, U-to-C RNA editing is present abundantly in hornworts (Kugita et al., 2003), and ferns (Wolf et al., 2004; Guo et al., 2015), but not in seed plants (Tillich et al., 2006). The *Selaginella* chloroplast genome has a particularly high number of C-to-U editing events, but no U-to-C editing (Hecht et al., 2011; Oldenkott et al., 2014). As a close relative of *Selaginella*, the research on the chloroplast genome of *Isoetes* is still limited to the phylogenetic analysis (Schafran et al., 2018; Wood et al., 2020; Pereira et al., 2021b). In addition, the research on RNA editing sites is limited to prediction using the PREPACT tool (Oldenkott et al., 2014), and lacks transcriptome data for verification.

In this study, we sequenced and compared the transcriptomes and chloroplast genomes of the six individuals (five species) which are distributed at an altitude between 100 and 4,300 m above sea level in China. Based on the generated dataset, we analyzed a total of six individuals’ chloroplast genomes and transcriptomes of *Isoetes* with the aim of (i) evaluating the structural features of the chloroplast genome, (ii) identifying RNA editing sites in chloroplast genomes of six individuals based on RNA-Seq data, and (iii) studying the genetic mechanism of its adaptation to high altitude.

## Materials and methods

### Sampling, DNA/RNA extraction, and sequencing

Plants were harvested from type localities whenever possible in China (Supplementary Table S1). Then, we collected the leaves of *I. yunguiensis*, *I. shangrilaensis*, *I. taiwanensis*, *I. sinensis*, and *I. hypsophila*, washed them with distilled water, fixed them in RNAlater solution (Takara, Dalian, China) immediately, and stored them in a −80°C freezer for DNA and RNA extraction. DNA quality and DNA concentration were measured on a NanoDrop 2000 (Supplementary Table S2).

We sequenced the transcriptomes and chloroplast genomes of the six individuals. Total genomic DNA was extracted using an extract Plant DNA kit (TIANGEN, China), while total RNA was isolated using the RNAiso Plus kit (TaKaRa, Dalian, China). Afterward, a paired-end library with an insert size of 350 bp was constructed using the Truseq Nano DNA HT Sample Prep Kit (Illumina, United States), and the RNA-sequencing library was generated using the VAHTS mRNA-seq v2 Library Prep Kit for Illumina^®^ (Vazyme, NR601).

### Chloroplast genome and transcriptome *de novo* assembly and annotation

Raw data were processed by removing linker sequences and removing low-quality reads at the Q20 cutoff, and subsequent analyses were based on these filtered high-quality sequences. The *de novo* assembly of the chloroplast genome was carried out using GetOrganelle with parameter settings as follows: ‘-R 15 -k 21,45,65,85,105 -F embplant_pt’ (Jin et al., 2020). Then, the wrong bases of the organelle genomes were corrected using BWA (Li and Durbin, 2010) and Pilon with default parameters. The chloroplast genome annotations were performed in GeSeq (Tillich et al., 2017). The tRNA genes were further verified using the tRNAscan-SE program (Schattner et al., 2005). We used Geneious (Kearse et al., 2012) to validate the annotated six chloroplast genomes by comparison with reference chloroplast genomes of *Isoetes nuttallii*, and *Isoetes cangae* (NCBI accession numbers: NC_038073, MG019394). Finally, the resulting chloroplast genome maps were drawn with Chloroplot (Zheng et al., 2020).

We assembled the transcriptomes by *de novo* assembly of high-quality RNA-Seq data using Trinity (Grabherr et al., 2011), followed by splicing of Trinity-obtained contigs into transcripts, after which only the longest transcripts in each cluster were selected as unigenes for subsequent analysis.

### Comparative genome analysis

Comparative genomics and visualization of six *Isoetes* chloroplast genomes were performed using mVISTA software (Frazer et al., 2004) with annotations of *I. taiwanensis* as a reference. The genes on the IR, SSC, and LSC boundaries were visualized using the tool IRscope (Amiryousefi et al., 2018) based on the annotation information.

### Identification of RNA editing sites

Geneious (Kearse et al., 2012) was used to map the RNA reads from each individual to their chloroplast genomes. Variants with <5× read depth and <2.5% of RNA reads mapped to a given fragment were excluded to address possible sequencing errors. RNA editing efficiency was calculated by dividing the edited reads by the total mapped reads.

### Phylogenetic analysis

To determine the phylogenetic relationships between *Isoetes* species, the CDS of chloroplast genome sequences and the transcriptome data were used to construct trees. For the phylogenetic tree constructed from chloroplast genome sequences, 72 protein-coding genes shared by 34 species were extracted. In addition, for the genes in the IR region, we only extracted one copy of them. The 28 completed chloroplast genome sequences were downloaded from the NCBI Organelle Genome Resource database. GenBank information for all of the chloroplast genomes used for the present phylogenetic analyses is found in Supplementary Table S3. For the phylogenetic tree constructed from transcriptome data, we first aligned the amino acid sequences of each single-copy gene orthologous using muscle with default parameters (Edgar, 2004). The alignment file of each orthologous gene was then concatenated into a super gene alignment, which was further trimmed to remove poorly aligned regions using trimal (Capella-Gutiérrez et al., 2009). Among them, the genome data of the two species *Selaginella moellendorffii* (Banks et al., 2011) and *Isoetes taiwanensis* (Wickell et al., 2021) have been published, so the genome protein file was used in the construction of the tree. *Selaginella moellendorffii* was set as the outgroup.

Maximum likelihood (ML) analysis was performed using the RAxML v 8.0.5 software package (Stamatakis, 2014) with 1,000 non-parametric bootstrap replicates. Bayesian Inference (BI) phylogenies were inferred using MrBayes 3.2.6(Ronquist et al., 2012) under JC + I + G model (2 parallel runs, 2,000,000 generations), in which the initial 25% of sampled data are discarded as burn-in.

### Orthologous gene identification and positive selection analysis

We used the branch-site model in the PAML (Yang, 2007) CODEML program to identify positively selected genes in the six *Isoetes* chloroplast genomes collected from China. The null model refers to the assumption that all branches evolve at the same rate, and the alternative model differs from the null model by allowing the foreground branches to evolve at different rates. In addition, the likelihood ratio test (LRT; Nielsen and Yang, 1998) was used to evaluate the statistical significance of each pair of nested models. We set *I. hypsophila* collected from high altitudes as the foreground branch in the branch-site model and others as the background branch.

The Reciprocal best hit (RBH) algorithm is the most commonly used algorithm based on the Basic Local Alignment Search Tool (BLAST; Altschul et al., 1997), which defines orthologous genes as sequences of a pair of genes from two genomes that are the best hits to each other (Moreno-Hagelsieb and Latimer, 2008). Next, we identified positively selected genes in the six transcriptomes based on orthologous genes by using the CODEML program’s branch-site model, where the foreground branch was set identically to the chloroplast genome. Only orthologous genes with *p* < 0.05 were considered positively selected genes; otherwise, orthologous genes were considered non-positively selected genes. KEGG enrichment analysis was performed using the OmicShare tools. These genes were annotated using eggNOG-mapper (Huerta-Cepas et al., 2017) in the eggNOG database.

## Results

### Transcriptome features and orthologous genes

Illumina pair-end sequencing produced 613,197,232 raw reads for six individuals, and 608,937,022 clean reads were obtained after removing low-quality reads and ambiguous nucleotides (Supplementary Table S4). Q30 and Q20 of clean reads were above 93% and 97%, respectively, which indicated that these data could be used in subsequent analysis. Based on Trinity assembly, extraction of the longest transcript yielded a total of 457,357 unigenes, and 2,798 single-copy orthologous genes were identified using RBH.

### Chloroplast genome features

**Figure 1:**
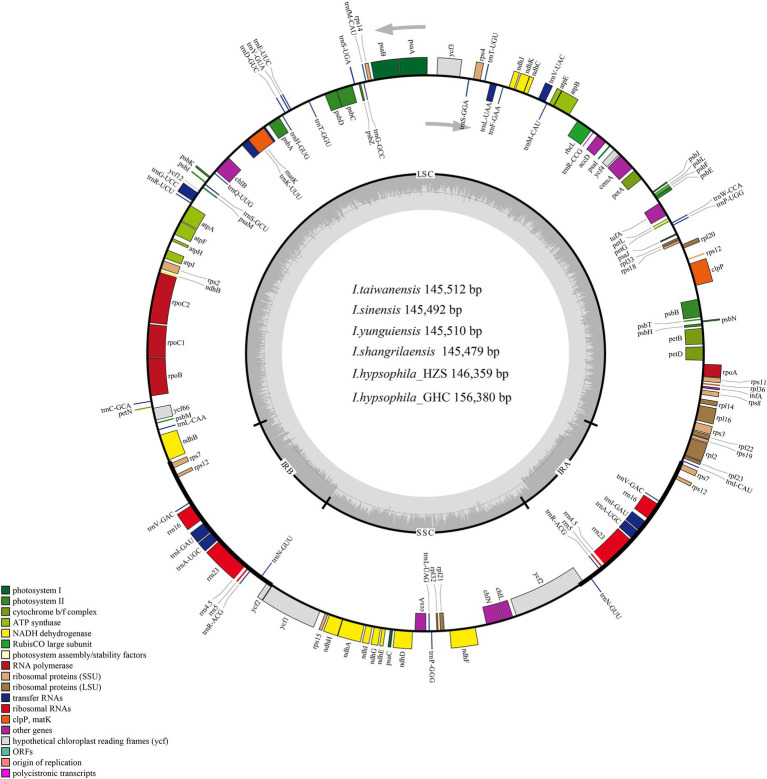
Chloroplast genome maps of *Isoetes* species. Genes belonging to different functional groups are color-coded. The darker grey in the inner circle shows the GC content, while the lighter grey shows the AT content.

The complete chloroplast genomes of the six *Isoetes* species ranged from 145,479 bp (*I. shangrilaensis*) to 146,380 bp (*I. hypsophila_*GHC), with 38%–38.1% GC content, which were composed of four regions, including LSC (91,740–91,880 bp) and SSC (27,218–27,272 bp) region separated by two IRs (13,207–13,691 bp; Figure 1; Table 1). A total of 135 genes were annotated from *Isoetes* chloroplast genome: 84 protein-coding genes, 8 rRNA genes, 36 tRNA genes, and 7 pseudogenes (Table 2). Among these genes, 24 were duplicated in the IR regions: 6 protein-coding genes, 10 tRNA, and 8 rRNA genes. There were 18 genes with introns, of which 16 had one intron, and two genes (*clpP*, *ycf3*) had two introns. Internal stop codons were observed in the CDS of 16 genes in *Isoetes*, except for *I. hypsophila* which *rps3* without internal stop codons. In addition, except for *rpoC1* and *rpoC2*, all genes contained a single internal stop codon. The *rpoC2* gene revealed three internal stop codons and observed two premature stop codons in *rpoC1*.

**Table 1:** The basic characteristics of the chloroplast genomes of six *Isoetes* individuals.

Features	*I. sinensis*	*I. taiwanensis*	*I. yunguiensis*	*I. shangrilaensis*	*I. hypsophila_*HZS	*I. hypsophila_*GHC
Genome size (bp)	145,506	145,512	145,510	145,479	146,359	146,380
LSC length (bp)	91,866	91,880	91,881	91,830	91,740	91,798
IR length (bp)	13,207	13,207	13,207	13,209	13,691	13,655
SSC length (bp)	27,226	27,218	27,215	27,231	27,237	27,272
Number of genes	135	135	135	135	135	135
Protein-coding genes	84	84	84	84	84	84
tRNA genes	36	36	36	36	36	36
rRNA genes	8	8	8	8	8	8
Pseudogene	7	7	7	7	7	7
Total GC content (%)	38.0	38.0	38.0	38.0	38.1	38.1
LSC	36.5	36.5	36.5	36.5	36.6	36.5
IR	48.0	48.0	48.0	48.0	47.8	47.8
SSC	33.4	33.4	33.4	33.3	33.5	33.5

**Table 2:** Gene annotation of the Isoetes chloroplast genomes.

Category	Group	Genes
Photosynthesis related genes	Rubisco	*rbcL*
	Photosystem I	*psaA*, *psaB*, *psaC*, *psaI*, *psaJ*, *psaM*
	Photosystem II	*psbA*, *psbB*, *psbT*, *psbK*, *psbI*, *psbH*, *psbM*, *psbN*, *psbD*, *psbC*, *psbZ*, *psbJ*, *psbL*, *psbE*, *psbF*
	ATP synthase	*atpA*, *atpB*, *atpE*, *atpF*^a^, *atpH*, *atpI*^e^
	Cytochrome b/f complex	*petA*, *petB*^a^, *petD*^a^, *petN*, *petL*, *petG*^e^
	Cytochrome csynthesis	*ccsA* ^e^
	Complex I of chloroplasts Chlorophyll biosynthesis	*ndhA*^a^, *ndhB*^a,d^ (×2), *ndhC*, *ndhD*^e^, *ndhE*, *ndhF*^e^, *ndhH*^e^, *ndhG*, *ndhJ*^e^, *ndhK*, *ndhI* *chlB*, *chlL*, *chlN*
Transcription and translation related genes	Transcription	*rpoA*^e^, *rpoB*^e^, *rpoC2*^e^, *rpoC1*^a,e^
	Ribosomal proteins	*rps2*, *rps3*^e^, *rps4*, *rps7*^c^ (×2), *rps8*, *rps11*, *rps12*^a,c^ (×2), *rps14*, *rps15*, *rps16*^d^, *rps18*, *rps19*, *rpl2*^a,d^, *rpl14*^e^, *rpl16*^a^, *rpl20*, *rpl21*^e^, *rpl22*, *rpl23*, *rpl32*, *rpl33*, *rpl36*
	Translation initiation factor	*infA* ^d^
RNA genes	Ribosomal RNA	*rrn16S*^c^ (×2), *rrn23S*^c^ (×2), *rrn4.5*^c^ (×2), *rrn5*^c^ (×2)
	Transfer RNA	*trnH-GUG*, *trnK-UUU*^a^, *trnQ-UUG*, *trnS-GCU*, *trnS-UGA*, *trnS-GGA*, *trnG-GCC*, *trnG-UCC*^a^, *trnR-UCU*, *trnR-ACG*^c^ (×2), *trnR-CCG*, *trnC-GCA*, *trnD-GUC*, *trnY-GUA*, *trnE-UUC*, *trnT-UGU*, *trnT-GGU*, *trnfM-CAU*, *trnL-CAA*, *trnL-UAA*^a^, *trnL-UAG*, *trnF-GAA*, *trnV-GAC*^c^ (×2), *trnV-UAC*^a^, *trnM-CAU*, *trnW-CCA*, *trnP-UGG*, *trnP-GGG*, *trnI-CAU*, *trnI-GAU*^a,c^ (×2), *trnA-UGC*^a,c^ (×2), *trnN-GUU*^c^ (×2)
Other genes	RNA processing	*matK* ^e^
	Carbon metabolism	*cemA*
	Fatty acid synthesis	*accD* ^d^
	Proteolysis Elongation factor Tu	*clpP* ^b^ *tufA* ^d^
	Conserved ORFs	*ycf1*, *ycf2*^c,d,e^ (×2), *ycf3*^b^, *ycf4*, *ycf12*, *ycf66*^a^

^a^Genes with one intron.

^b^Genes with two introns.

^c^Two gene copies in IRs.

^d^Pseudogene.

^e^Genes with internal stop codon.

### Comparative genome analysis

**Figure 2:**
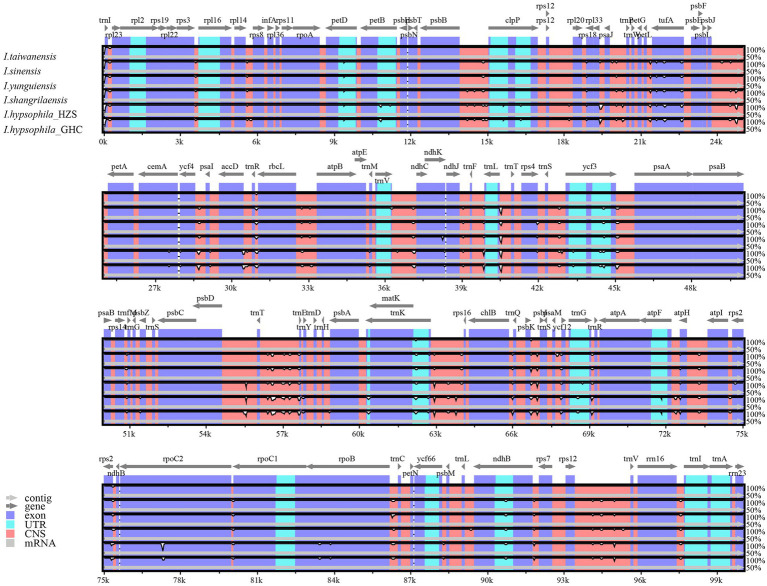
Comparison of the borders of LSC, SSC, and IR regions among six *Isoetes* chloroplast genomes.

To investigate the extent of divergence in the sequences of the chloroplast genomes of the genus *Isoetes*, the six *Isoetes* chloroplast genome sequences were aligned by using the mVISTA, with the *I. taiwanensis* annotation as a reference. The results of sequence alignment revealed intragenus sequence differences in the chloroplast genome, and the results showed that the highly differentiated regions were mainly located in intergenic regions, such as *trnR*-*trnN*, and there were also variant regions in the coding regions such as *rpoC2*, *rpoB*, *ycf1,* and *ycf2* (Figure 2). Overall, the high degree of gene order conservation was detected in the six chloroplast genomes, indicating evolutionary conservation at the genome scale.

**Figure 3:**
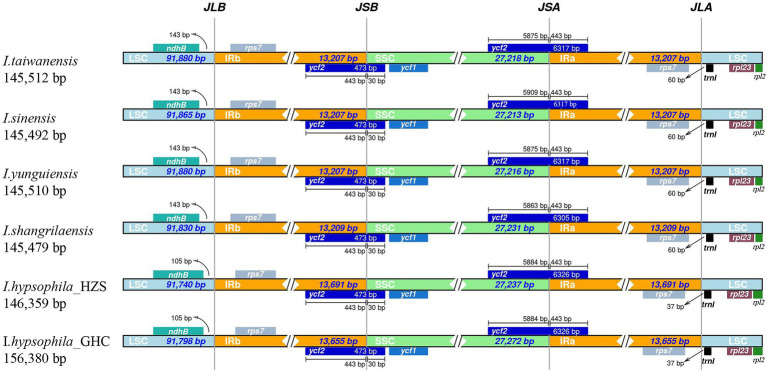
Global alignment of chloroplast genomes of six *Isoetes*, with the *I. taiwanensis* genome as the reference. Gray arrows indicate the direction of gene transcription. Red blocks indicate conserved non-coding sequences (CNS), and blue blocks indicate conserved genes. The *y*-axis represents the percent identity within 50%–100%.

A comprehensive comparison of the IR-SSC and IR-LSC boundaries of the chloroplast genomes of the six *Isoetes* individuals is presented in Figure 3. The genes *ndhB*, *rps7*, *ycf2*, and *rpl23* are located at the junction of the LSC/IRa, IRa/SSC, SSC/Irb, and IRb/LSC borders, respectively. The *ycf2* gene is located at the junction of the IRb/SSC and the border has moved toward the SSC region because there are 30 bp sequences situated at SSC region and the *trnI* gene is located in the LSC, 37–60 bp from the IRa/LSC boundary. Overall, the chloroplast genomic structure of the six *Isoetes* individual is concordant, while differences in the lengths of four regions lead to six genome sizes ranging from 145,479 to 146,380 bp.

### RNA editing analysis

To identify RNA editing sites, all the transcriptome reads were mapped to the chloroplast genomes using Geneious software. The type, position, and editing efficiency of the editing sites are presented in Supplementary Table S5. The RNA editing analyses revealed the presence of 291 (*I. hypsophila_*GHC) to 579 (*I. taiwanensis*) RNA editing sites in six individuals. All editing types appearing in internal stop codons are U-to-C editing, which results in codon changes from stop codons (UAA, UGA) to glutamine (CAA) or arginine (CGA).

**Figure 4:**
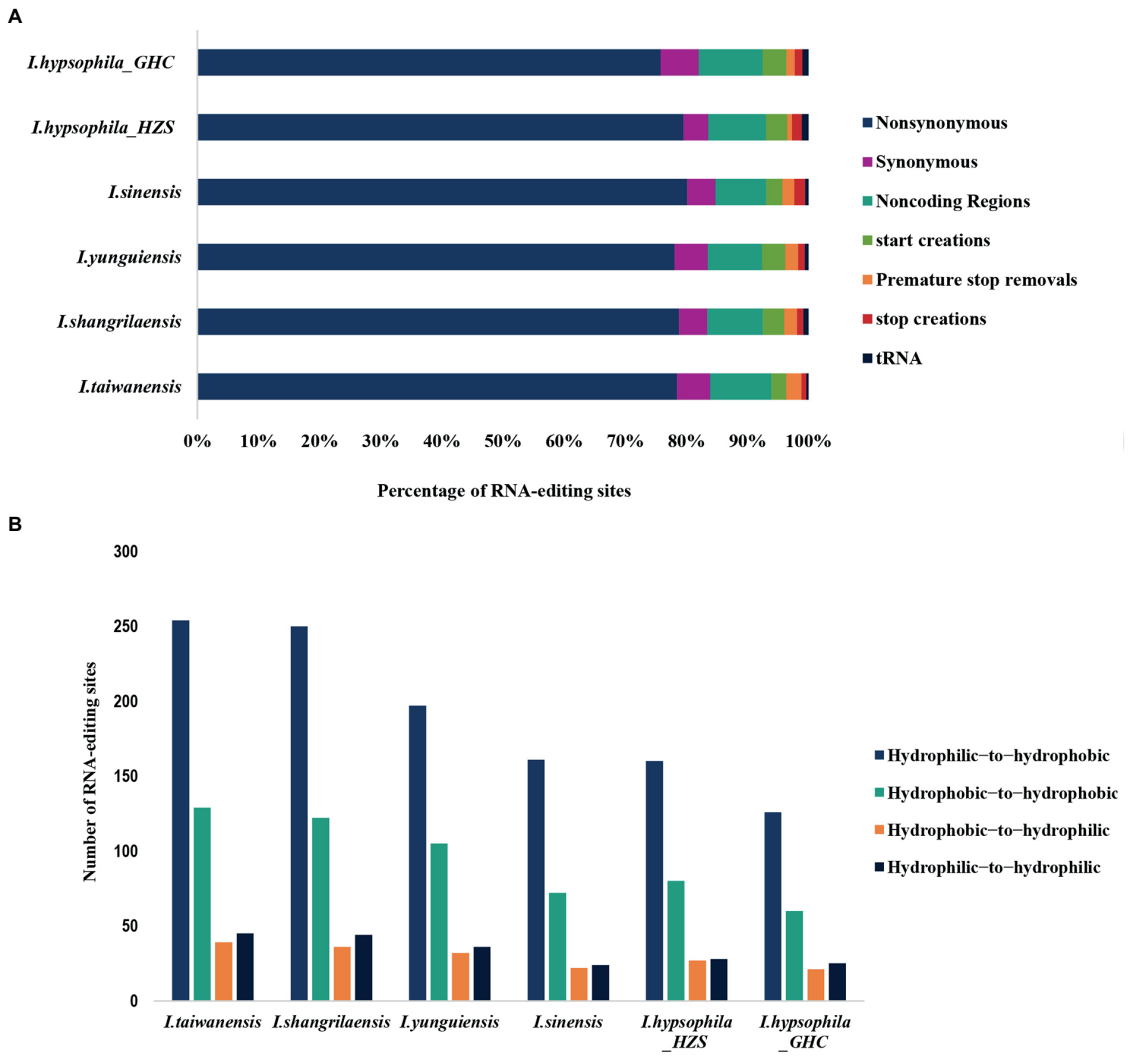
**(A)** Percentage of specific sequence modifications produced by RNA editing in sampled individuals. Histograms represent 100% of RNA edits detected. **(B)** Comparison of edit sites that lead to a change in hydrophobicity/hydrophilicity of the resulting amino acid *via* non-synonymous RNA editing.

We found that the majority (nearly 78%) of edits in coding regions resulted in non-synonymous amino acid changes in six *Isoetes* individuals, and <5% of edits were synonymous rather than coding regions (UTRs and introns) and tRNAs have also found some RNA editing sites (Figure 4A). In addition, we investigated the effect of RNA editing on the hydrophobicity of the encoded amino acid, with the vast majority of non-synonymous RNA editing converting codons for hydrophilic amino acids to codons for hydrophobicity (Figure 4B).

### Phylogenetic relationships and positive selection analysis

**Figure 5:**
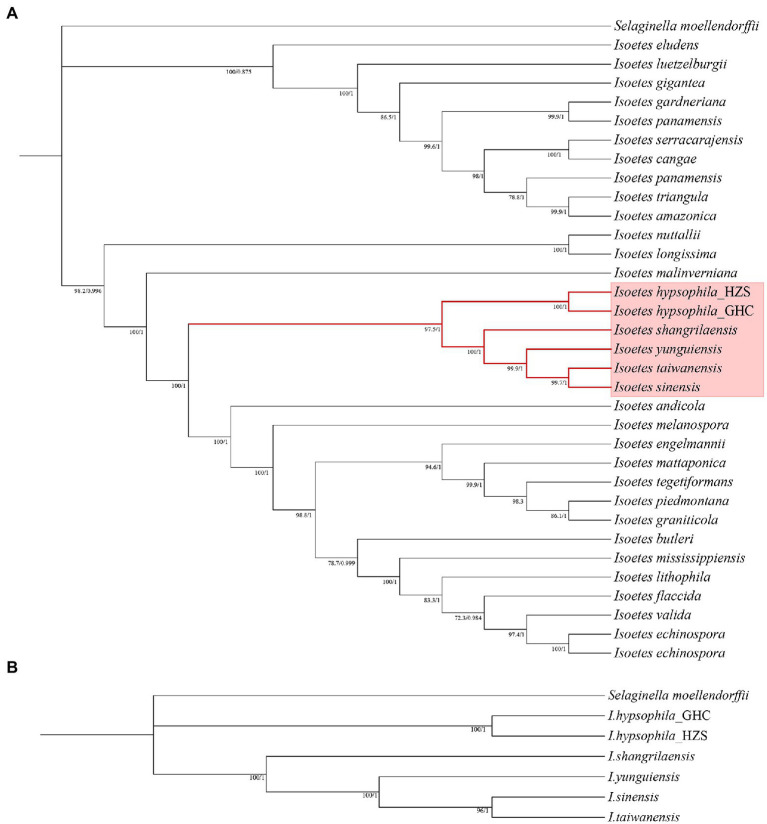
**(A)** Phylogenetic tree constructed using protein-coding regions of the chloroplast genomes. **(B)** Phylogenetic tree constructed using transcriptome data.

In order to reconstruct a phylogeny for further selection analyses, two phylogenetic trees were constructed using transcriptome data and the CDS from the chloroplast genomes, respectively. Although the long evolutionary history of *Isoetes*, with its split from the closest extant relatives in the Devonian, is a confounding factor in establishing phylogenetic relationships in this genus, our chloroplast-based phylogeny analysis may contribute to understanding the diversification of *Isoetes* and provide a highly robust framework for investigating the evolutionary history of the genus. The backbone of the phylogenetic reconstruction and most of the clades agree with previous studies by Larsén and Rydin (2016) and Pereira et al. (2017). For species from China, we found all phylogenetic trees exhibited similar clustering, which showed two different evolutionary branches. The resulting phylogenetic trees demonstrated that alpine species *I. hypsophila* were located on one branch, whereas other species were located on another branch (Figure 5).

**Figure 6:**
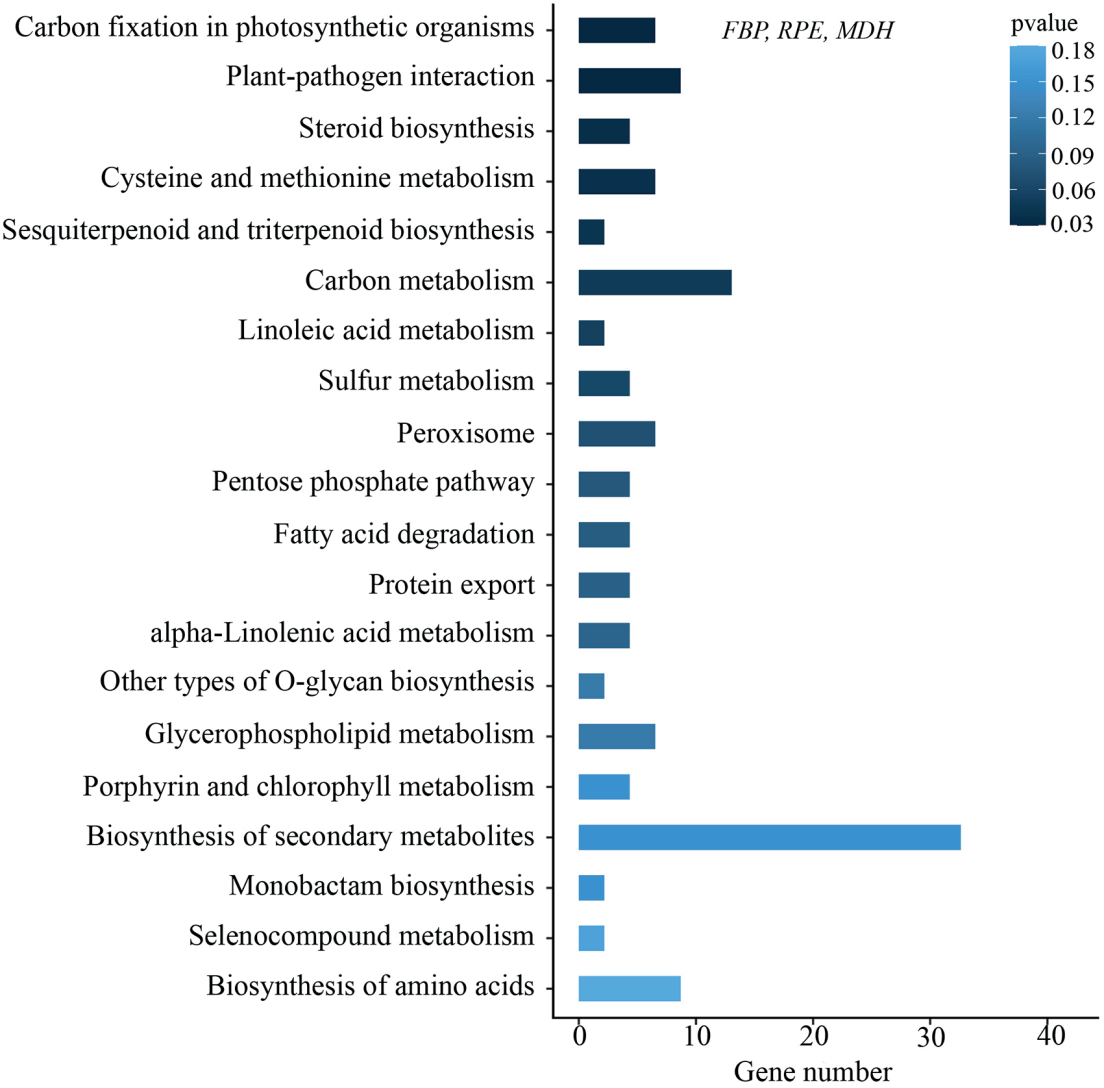
Distribution of KEGG classifications of PSGs in *I. hypsophila*.

For the chloroplast genome, a total number of 86 common genes were involved in the positive selection analysis, of which 16 genes with internal stop codons were corrected back to normal coding sequences before analysis (Supplementary Table S6). The branch-site model detected only one gene (rps3) containing sites that had been subject to positive selection (Table 3). In addition, a total of 2,798 single-copy orthologs were identified in transcriptome data among the six individuals. Of these genes, 46 positively selected genes (PSGs) were annotated and enriched on the KEGG pathway (Supplementary Table S7). The top 20 clusters of the KEGG functional analyses with their representative enriched pathway are shown in Figure 6. The pathway “carbon fixation in photosynthetic organisms” was enriched, in which three PSGs encode fructose-1,6-bisphosphatase (*FBP*), ribulose-phosphate 3-epimerase (*RPE*), and malate dehydrogenase (*MDH*).

**Table 3:** Positively selected genes and sites detected in the chloroplast genomes of Isoetes species.

Gene name	Positive sites	*p*-value
rps3	96S;123Y	0.001423015

## Discussion

### Chloroplast genome features

The whole chloroplast genome sequences newly obtained herein for six individuals are not only very similar in size (145,479–146,380 bp; Table 1), but also in overall structure, gene order, and content (Figure 2). These findings are consistent with a previous study on *Isoetes*, which showed that the chloroplast sequences and gene arrangements were conserved (Pereira et al., 2021a), Although the sequence is conserved, there are still some features worth discussing. Both large-scale studies (Shaw et al., 2014) and specific case studies (Ye et al., 2018; Alwadani et al., 2019) have demonstrated higher differences in non-coding regions, some of the non-coding sequences we detected had hypervariable regions, such as *psbB-clpP*, *psbJ-petA*, *trnK-rps16*, *rpoB-trnC*, and *rps12-trnV*. Furthermore, we found greater differences in chloroplast genome sequences between the high-altitude species *I. hypsophila* and other *Isoetes* species, such as *rpoC2* and *rpoB*, suggesting that altitude may drive genetic differentiation, as evidenced by other studies (Liu et al., 2020). These newly discovered regions can be used for subsequent species identification and provide additional phylogenetic information.

In terms of the GC content of the six *Isoetes*, the total GC content of the complete chloroplast genome is ~38%, similar to the previously published (Pereira et al., 2021a). In general, the effect of GC content on chloroplast genome stability is more pronounced because GC base pairs and AT base pairs are thermodynamically different in stability (Yang et al., 2021). Among the LSC, SSC, and IR regions, the IR regions have the highest GC content, followed by the LSC and SSC regions. The IR region has the highest GC content among the four regions, probably because of the high G/C content of rRNAs in this region.

### RNA editing sites

In plants, RNA editing plays an irreplaceable role in growth and development. RNA editing has been observed in the chloroplasts of extant descendants of early land plants other than liverworts and mosses. In angiosperm chloroplasts, RNA editing is mostly restricted to a C-to-U conversion, and the conversion occurs at about 30 different positions (Yu et al., 2020), whereas the range of variation in RNA-editing sites is even more remarkable in hornworts and fern chloroplasts. It is rare in the moss *Physcomitrella patens*, which holds only 11 C-to-U edit sites (Rüdinger et al., 2009), and is completely absent from the liverwort *Marchantia polymorpha* (Rüdinger et al., 2008), whereas it is most abundant in hornworts and fern, with over 300 different positions (Stern et al., 2010). The reduced number of PPR genes and absence of RNA editing in marchantiid liverworts are most probably secondary losses, as the organellar RNA editing and plant-specific extensions of PPR genes were also found in jungermanniid liverworts (Rüdinger et al., 2008; Zhang et al., 2020). Our analyses revealed obvious differences in the number of RNA editing sites in the chloroplast genomes of the genus *Isoetes*, that is, the range of number from 291 in *I. hypsophila*_GHC to 579 in *I. taiwanensis* (Supplementary Table S5). Interestingly, we found that there were large differences in the number of RNA editing sites of the same species in different populations, including 291 RNA editing sites in *I. hypsophila*_GHC, and 354 RNA editing sites in *I. hypsophila*_HZS. This difference also exists in the RNA editing of the mitochondria of the three ecotypes of *Arabidopsis thaliana* (Zehrmann et al., 2008). This situation may suggest that the environment has a greater influence on the RNA editing site, and subsequent studies can focus on this aspect. The pentatricopeptide repeat (PPR) is a family of RNA binding proteins involved in specific RNA processing events such as RNA editing, translation initiation, and transcript processing (Ichinose and Sugita, 2018). Previous studies have shown that chloroplast RNA editing abundance is positively correlated with the PPR gene family (Rudinger et al., 2012; Xu et al., 2018). Taken together, these species with a large number of RNA editing sites are early landing plants. Through RNA editing, PPR proteins can act as “repair” factors, alleviating DNA damage caused by increased UV exposure during adaptive landing (Zhang et al., 2020).

A pseudogene is rendered non-functional through the introduction of stop codons predominantly in the chloroplast genome. Conversely, in mosses and ferns, genes that contain internal stop codons can still make proteins function properly because U-to-C RNA editing can convert translation internal stop codons (such as the UAA termination signal) into CAA triplet encoding the amino acid glycine. In addition, there are some special cases, such as in Selaginellaceae, because the absence of U-to-C RNA editing cannot eliminate the internal stop codons, so some genes become nonfunctional pseudogenes (Gerke et al., 2020). However, *Isoetes*, which is the closest relative of Selaginellaceae, can convert internal stop codons to functional amino acids. This indicates that these two species that diverged from a common Lycopsida ancestor may evolve different mechanisms to achieve the same ends.

For each species, roughly 78% of the RNA-editing events did not involve a non-synonymous amino acid change in start or stop codons (Figure 4A). Furthermore, we found that the proportion of *Isoetes* RNA editing sites in non-coding regions and tRNAs are similar (Figure 4A), while we observed that RNA editing greatly increased the proportion of hydrophobic amino acids (Figure 4B), and the hydrophobicity has long been considered as one of the primary drivers of protein folding and protein function (Moelbert et al., 2004; Li et al., 2016). Therefore, we speculate that the increase in the hydrophobicity due to a large number of RNA editing may facilitate the translation of mRNA into polypeptides with folded structures at the appropriate locations, which are often necessary for proteins to form functional three-dimensional (3D) structures (Yura and Go, 2008).

### Adaptive evolution

With the recent development of genome technology, the investigation of genome-wide molecular mechanisms of high-altitude adaptation has attracted great attention in the last few years (Chen et al., 2019; Hao et al., 2019; Zhang et al., 2021). Even though whole nuclear genome sequencing allows investigation of the impact of selection events at the genome-wide level, it is expensive and not easily available. On the contrary, transcriptome sequencing has been described as a powerful method for genome-wide analysis of high-altitude adaptation and is cheaper and easier available than the nuclear genome. Additionally, QTP is the highest plateau in the world, with an extreme environment of hypoxia, low temperature, and strong solar radiation (Mao et al., 2021). Solar radiation is one of the main stresses faced by alpine plants, and chloroplasts, as the site for photosynthesis, may have acquired adaptive strategies to strong solar radiation (Yoshida et al., 2019). Thus, both transcriptome and chloroplast genomes represent a great system to study the footprint of alpine plants in adaptation to QTP. Nevertheless, previous studies have either focused on transcriptome or chloroplast genome to study altitude adaptation, while the feasibility of integrating transcriptome and chloroplast genome to uncover the adaptive mechanisms to QTP remains less explored. Thus, in this study, we integrated the chloroplast genome and transcriptome data to explore the molecular mechanism of the adaptation of alpine plants to the high altitude of QTP, and we expect our research can provide a reference for future genome-wide studies on the adaptive evolution of alpine plants on the QTP.

Alpine species on the QTP have to evolve to have a high ability to adapt to extremely harsh environments (Thompson et al., 2000; Norsang et al., 2011). Intense UV radiation is a major environmental stressors for plants, and recent studies have revealed candidate genes for plateau adaptability, mainly associated with UV radiation in a variety of plants (Mao et al., 2021). We anticipated that some genes in the chloroplast genome of *I. hypsophila* might have undergone adaptive evolution to adapt to the alpine environment, although overall genome size, structure, and gene number have changed slightly. Using *I. hypsophila* as the foreground, the branch-site model detected rps3 as the possible positively selected gene (PSG). The rps3 (Ribosomal protein S3) gene encodes ribosomal small subunit protein 3, which belongs to the ribosomal protein S3P family and is a part of the ribosomal 40s subunit (Korovesi et al., 2018), and plays important roles in repairing damaged DNA and apoptosis (Dong et al., 2017; Kim et al., 2018). The branch-site model detected two positively selected sites (96S,123Y) of rps3 in *I. hypsophila*, which were probably involved in the protection of *I. hypsophila* from strong UV radiation, drought, and other stressful environments in higher altitudes.

In addition, our study revealed many nuclear-encoded genes involved in high-altitude adaption and the genes may play an important role in the adaption to the high-altitude environment of QTP. Through the positive selection analysis of transcriptome data, a total of 46 positive selection genes were enriched in the KEGG pathway, and the “carbon fixation in photosynthetic organisms” pathway was significantly enriched (Figure 6), of which all three detected PSGs encode proteins with functions related to photosynthesis (*NADP-MDH*, *RPE*, and *FBP*). In plants, the *NADP-MDH* is the key enzyme controlling the malate valve, which plays a role in the export of reducing equivalents in photosynthesizing chloroplasts (Vaseghi et al., 2018). The RPE is an enzyme in the chloroplast-localized oxidized pentose phosphate pathway that is essential for both the Calvin cycle and the reverse pentose phosphate pathway (Liu et al., 2015). The FBPase is a rate-limiting enzyme in the carbohydrate metabolism and the Calvin cycle, which plays a pivotal role in carbohydrate biosynthesis (Lee et al., 2008). A previous study reported that the increased cell growth rate and enhanced photosynthetic activity could be achieved by increasing the levels of FBP aldolase in Anabaena (Ma et al., 2007). Given that high elevations are characterized by high levels of UV radiation, which pose a serious challenge to plant photosynthesis and may lead to positive selection of the related genes (Li et al., 2020; Moutinho et al., 2020), it is possible that these three PSGs were driven by natural selection in high elevation environments and may contribute to high-altitude adaptation in *Isoetes* species.

## Data availability statement

The data presented in the study are deposited in the NCBI repository, accession number: SRR18531157, SRR18531155, SRR18531156, SRR18531154, SRR18531159, SRR18531158, OM283821, OM283822, OM283818, OM283820, OM283819, and OM283817.

## Author contributions

XinL and XiaL designed the study. YY and XY assembled, annotated, and analyzed the plastomes. PW and YY detected RNA editing sites. YY drafted the manuscript. CL and ZC revised the manuscript. All authors contributed to the article and approved the submitted version.

## Funding

This work was supported by a grant from the National Natural Science Foundation of China (31170203).

## Conflict of interest

The authors declare that the research was conducted in the absence of any commercial or financial relationships that could be construed as a potential conflict of interest.

## Publisher’s note

All claims expressed in this article are solely those of the authors and do not necessarily represent those of their affiliated organizations, or those of the publisher, the editors and the reviewers. Any product that may be evaluated in this article, or claim that may be made by its manufacturer, is not guaranteed or endorsed by the publisher.
