# Supplementary material for: Comparative chloroplast genome and transcriptome analysis on the ancient genus Isoetes from China
Source: Front Plant Sci. 2022 Jul 29;13:924559. doi: 10.3389/fpls.2022.924559 (PMC9372280; doi:10.3389/fpls.2022.924559)
Supplement: Supplementary file 1 [file Data_Sheet_1.ZIP › Supplementary materials/Table S1.docx]

Table S1 Locality, and Genbank accession number of the six newly sequenced species.

| Species | Voucher | Locality | Elevation  (m) | GenBank Accession  (Transcriptomes) | GenBank Accession  (Chloroplast genomes) |
| --- | --- | --- | --- | --- | --- |
| *I.sinensis*  *I.taiwanensis*  *I.yunguiensis*  *I.shangrilaensis*  *I.hypsophila_*HZS  *I.hypsophila_*GHC | *I.sinensis*_JD | Jiande, Zhejiang Province, China | 134 | SRR18531157 | OM283821 |
|  | *I.taiwanensis*_TW  *I.yunguiensis*_PB  *I.shangrilaensis*_XJS  *I.hypsophila_*HZS  *I.hypsophila_*GHC | Taiwan, China  Pingba, Guizhou Province, China  Shangri-La, Yunnan Province, China  Daocheng, Sichuan Province, China  Gaohan Village, Yunnan Province, China | 700  1280  3305  4348  3278 | SRR18531155  SRR18531156  SRR18531154  SRR18531159  SRR18531158 | OM283822  OM283818  OM283820  OM283819  OM283817 |
