# Supplementary material for: Comparative chloroplast genome and transcriptome analysis on the ancient genus Isoetes from China
Source: Front Plant Sci. 2022 Jul 29;13:924559. doi: 10.3389/fpls.2022.924559 (PMC9372280; doi:10.3389/fpls.2022.924559)
Supplement: Supplementary file 1 [file Data_Sheet_1.ZIP › Supplementary materials/Table S2 .docx]

Table S2. The quantity and quality for DNA and RNA

|  |  | *I.*  *sinensis* | *I.*  *taiwanensis* | *I.*  *yunguiensis* | *I.*  *shangrilaensis* | *I.*  *hypsophila_*HZS | *I.*  *hypsophila_*GHC |
| --- | --- | --- | --- | --- | --- | --- | --- |
| RNA | density (ng/μL) | 318 | 206 | 258 | 405 | 220 | 854 |
|  | volume (ul) | 22 | 23 | 25 | 46 | 18 | 24 |
|  | Total (ug) | 6.996 | 4.738 | 6.45 | 18.63 | 3.96 | 20.496 |
|  | evaluation | A | A | A | A | B | A |
| DNA | density (ng/μL) | 18.073 | 2.489 | 23.28 | 35.702 | 18.114 | 14.284 |
|  | volume (ul) | 55 | 55 | 55 | 55 | 55 | 55 |
|  | Total (ug) | 0.99402 | 0.1369 | 1.2804 | 1.96361 | 0.99627 | 0.78562 |
|  | evaluation | A | A | A | A | A | A |
