# Supplementary material for: Comparative chloroplast genome and transcriptome analysis on the ancient genus Isoetes from China
Source: Front Plant Sci. 2022 Jul 29;13:924559. doi: 10.3389/fpls.2022.924559 (PMC9372280; doi:10.3389/fpls.2022.924559)
Supplement: Supplementary file 1 [file Data_Sheet_1.ZIP › Supplementary materials/Table S4.docx]

Table S4. Statistical summary of Isoetes transcriptome for sequencing and de novo assembling

| Species | Total number of  raw reads (M) | Total number of  clean reads (M) | Q20 of clean data (%) | Q30 of clean data (%) | Total number  of unigenes |
| --- | --- | --- | --- | --- | --- |
| *I.sinensis*  *I.taiwanensis*  *I.yunguiensis*  *I.shangrilaensis*  *I.hypsophila_*HZS  *I.hypsophila_*GHC | 97.669906 | 97.016976 | 97.7 | 93.3 | 73462 |
|  | 77.014614  110.69698  116.111716  107.3686  104.335416 | 76.77044  109.89104  115.207024  106.373342  103.6782 | 98.1  97.4  97.5  97.6  97.6 | 94.6  92.6  92.8  93  93.1 | 64318  58322  85613  112948  85813 |
